# Compression Behavior and Textures of Ti57-Nb43 Alloy at High Temperatures

**DOI:** 10.3390/ma16227116

**Published:** 2023-11-10

**Authors:** Máté Szűcs, Viktor Kárpáti, Tamás Mikó, László S. Tóth

**Affiliations:** 1 Institute of Physical Metallurgy, Metal-Forming and Nanotechnology, University of Miskolc, Egyetemváros, 3515 Miskolc, Hungary; mate.szucs@uni-miskolc.hu (M.S.); femkv@uni-miskolc.hu (V.K.); femmiko@uni-miskolc.hu (T.M.); 2 Wigner Research Centre for Physics, High Energy Experimental Particle and Heavy Ion Physics, 1121 Budapest, Hungary; 3 Laboratory of Excellence on Design of Alloy Metals for Low-Mass Structure (Labex-DAMAS), Université de Lorraine, 57070 Metz, France; 4 Laboratoire d’Etude des Microstructures et de Mécanique des Matériaux (LEM3), Université de Lorraine, CNRS, ENSAM-Metz, 57070 Metz, France

**Keywords:** titanium–niobium alloy, stress–strain curve, high temperature, simulation, texture, strain rate sensitivity

## Abstract

The mechanical behavior, microstructures, as well as the crystallographic textures of the Ti57-Nb43 alloy were investigated on cylindrical specimens compressed at high temperatures, in the range of 700–1000 °C, and strain rates between 0.001 and 1.0 s^−1^. Hardening, followed by softening behaviors, were observed as a function of strain due to the occurrence of dynamic recrystallization/recovery in hot deformation. The modified five-parameter Voce-type equation described well the stress–strain curves, but, for the present alloy, it was also possible with only four parameters. A new two-variables polynomial function was employed on the four parameters that described well the flow curves as a direct function of temperature and strain rate. It permitted the reduction in the number of parameters and had the predictive capacity for the flow stress at any temperature, strain, and strain rate in the investigated range. The crystallographic textures were similar at all temperatures, with an increase in intensity from 900 °C. The textures could be characterized by a double <100> and <111> fiber and a unique component of (001) <110>, the latter inherited from the initial hot-rolling texture. Viscoplastic polycrystal self-consistent deformation modeling reproduced the measured textures showing that dynamic recrystallization did not alter the development of the deformation texture, only increased its intensity.

## 1. Introduction

Titanium–niobium alloys are excellent materials for producing superconducting magnets [1], and are widely used in nuclear magnetic resonance spectroscopy, in magnetic resonance imaging, or in particle accelerators. Therefore, it is important to examine their physical properties. Among them, forming properties are crucial for structural applications. The mechanical behavior can be readily examined by compression testing, which does not require sample grips, and larger strains can be reached before fracture, compared with tensile testing.

It is also important to describe the stress–strain behavior by analytical functions for the purpose of engineering design of the forming processes. There are several advanced formulas that are frequently used to obtain the stress as a function of plastic strain. The Johnson–Cook (‘J-C’) function includes three components that describe the effect of strain, strain rate, and temperature [2]:

(1) $$\sigma_{F}\left(\varepsilon ,\dot{\varepsilon },T\right)=\left(A+B\varepsilon^{n}\right)\cdot \left(1+Cln{\dot{\varepsilon }}^{*}\right)\cdot \left[1-{\left(T^{*}\right)}^{m}\right],$$

where $\sigma_{F}$ is the flow stress, ε is the strain, ${\dot{\varepsilon }}^{*}\;$ is the normalized strain rate (${\dot{\varepsilon }}^{*}=\dot{\varepsilon }/{\dot{\varepsilon }}_{ref}$), *T* is the normalized temperature ($T^{*}=\left(T-T_{ref.}\right)/\left(T_{m}-T_{ref.}\right)$), and *A*, *C, n*, *m*, $T_{ref.}$, and $T_{m}$ are the parameters ($T_{m}$: melting point, ref.: reference value). The J-C approach has seven parameters, and it is preferred to be used for metals whose flow curve shows a constant hardening behavior.

Similarly, Hensel and Spittel [3] developed a model that describes the flow stress of the material in terms of strain, strain rate, and temperature (‘H-S’ model), but with the capacity of capturing not only hardening, but the softening stage as well:

(2) $$\sigma_{F}\left(\varepsilon ,\dot{\varepsilon },T\right)=\sigma_{0}\cdot A_{1}e^{-m_{1}T}{\cdot A}_{2}\varepsilon^{m_{2}}\cdot A_{3}{\dot{\varepsilon }}^{m_{3}},$$

where $\sigma_{0}$ is the initial flow stress, and $A_{1}$, $A_{2}$, $A_{3}$, $m_{1}$, $m_{2}$, and $m_{3}$ are material parameters; the H-S model contains six, originally. It is widely used, and relatively efficient; see, for example, Mehtedi et al. [4], who applied this model for hot torsional deformation of aluminum alloys.

A mathematically more efficient constitutive equation was developed by Nguyen et al. [5], who proposed a modified Voce-type constitutive equation, which is also able to capture both hardening and softening:

(3) $$\sigma_{F}\left(\varepsilon \right)=\sigma_{0}+q_{1}\left(1-\exp\left(-c_{1}\varepsilon \right)\right)+q_{2}\left(1-\exp\left(c_{2}\varepsilon \right)\right),$$

where there are five parameters only ($\sigma_{0}$, $q_{1}$, $c_{1}$, $q_{2}$, $c_{2}$), although the strain rate and the temperature do not appear directly in the formula, only the strain does. A first application of Equation (3) on the Ti-6Al-4V alloy showed excellent reproduction of the experimental compression curves at high temperatures [5]. Of course, all parameter values were depending on the temperature (the strain rate was not varied). Then, an approximation of each parameter by a second-order polynom permitted explicit dependence on temperature, with a slight decrease in the fitting quality. In this way, the final number of coefficients was 19, and this was only for strain and temperature dependence.

Ebrahimi et al. [6] developed a mixed approach, using direct stress–strain formulas, together with the Zener–Hollomon relation (also called Arrhenius formula), the latter for considering the strain rate and temperature dependence. Their modeling uses two formulas: one up to the peak stress, and another after the peak stress, which includes the saturation stress as well. The number of parameters is only nine in this approach. The application is carried out on high-temperature compression of Ti-IF steel, with good results. Nevertheless, it is physically difficult to justify the use of two strain-hardening functions, as it implies the assumption of an instantaneous change in the microstructure processes at the peak stress.

None of the above presented modeling was used so far for studying the stress–strain behavior of Ti-Nb alloys in compression. Instead, only the Zener–Hollomon relation was employed for the construction of stress–strain curves, in the following form:

(4) $$\dot{\varepsilon \;}={A\left[\sinh\left(\alpha \sigma_{F}\right)\right]}^{n}\exp\left(-\frac{Q}{RT}\right),$$

where *Q* is the activation energy, *R* is the universal gas constant, and *A*, *α*, and *n* are parameters. Equation (4) contains the Zener parameter:

(5) $$Z=\dot{\varepsilon \;}\exp\left(\frac{Q}{RT}\right)\;\;,$$

which allows us to rewrite the Zener–Hollomon relation in the following form:

(6) $$\sigma_{F}=\frac{1}{\alpha }ln\left\{{\left(\frac{Z}{A}\right)}^{\frac{1}{n}}+\sqrt{{\left(\frac{Z}{A}\right)}^{\frac{2}{n}}+1}\right\},$$

The fundamental difference of this constitutive law from the above presented ones is that it does not contain the strain as a variable, only the strain rate and the temperature, through the Z parameter. Nevertheless, it is still possible to build a stress–strain relation, because the parameters are dependent on the stress value ($\sigma_{F}$), so depend on the strain as well. Such analysis was carried out by Shi-feng et al. [7] on a TC17 (Ti-5Al-2Sn-2Zr-4Mo-4Cr) titanium alloy, and also by Sun et al. [8] on the Ti-37 at.%Nb alloy. There is, however, a major difficulty in these works to find the parameters of the Zener–Hollomon formula. Namely, a formula for the activation energy, *Q*, is developed by Sun et al. [8], which is applied for a single value of the flow stress; the peak stress, therefore, its possible dependence on the stress-evolution is neglected. This is physically questionable, because it is exactly at the peak stress where there must be a change in the operating microstructure mechanisms, which necessarily should have different activation energies. Wan et al. [9], who examined the Ti-37 at.%Nb alloy in high-temperature compression, reported that the relationship between *Z* and $ln\left(\dot{\varepsilon \;}\right)$ and *1/T* does not satisfy a linear relationship when the deformation temperature is in the range of 790–940 °C for strain rates between 0.001 s^−1^ and 10 s^−1^; therefore, the Arrhenius equation is questionable.

Wan et al. [9] also applied a new computation technique, called the ‘GA-LSSVM’ algorithm, which was initially developed in the agriculture industry [10]. This technique uses non-linear mapping with artificial intelligence, without constitutive law, by dividing the measured data sets into ‘known’ and ‘unknown’ categories and predicting in this way the whole data set. While it was successfully used for the compression of the Ti-37 at.%Nb alloy at high temperatures, its use in metallurgical engineering is complex, while not leading to better results than using a constitutive law (mean relative error was 2.74%).

There are limited studies on the plastic behavior of Ti-Nb alloys at high temperatures, while there are comprehensive works on pure Ti [11] and pure Nb [12]. High Nb content is required for superconductivity and magnetic applications. Only Sun et al. [8] and Wan et al. [9] examined such alloys: in both studies, the Ti-37 at.%Nb alloy was investigated.

The above short analysis of the state of the art in stress–strain curve modeling of high temperature compression of metals shows that it is more promising to employ constitutive equations that contain strain as a direct variable, compared with the Arrhenius-type indirect approach.

Strain hardening/softening can be also produced by the evolution of the crystallographic texture of the material during plastic deformation. Hard texture components increase the yield stress while weaker ones reduce it. This effect can be examined by suitable polycrystal modeling, like the viscoplastic self-consistent (VPSC) model [13]. For example, it was observed in torsion of Mg that the initial strain hardening stage was due to the evolution of the texture, which could be modeled by the VPSC approach, without introducing any intrinsic hardening into the simulation [14].

In the present study, the Voce-type modified constitutive equation proposed by Nguyen et al. [5] was used for describing the hot compression stress–strain curves of Ti57-Nb43 at high temperatures, from 700 to 1000 °C, at four strain rates. It is shown that, instead of five, four parameters only are enough for a good reproduction of the experimental data. With the help of new polynomial functions, the parameters were explicitly expressed as a function of the temperature and strain rate. Good agreements have been reached between the modeled and experimental data in the range of 800–1000 °C and 0.01 to 1 s^−1^ testing, with only a 1.48% general average relative error.

It is also shown in the present work that the strain hardening behavior is not influenced by the texture evolution in the present Ti-Nb alloy. The observed softening in the experiment is the result of dynamic recrystallization, which could be confirmed by detailed examination of the microstructure. It is important to identify the mechanisms that control the strength of the material. This is the first study where the relation between strength and texture evolution is examined in compression or rolling of Ti-Nb alloys.

During compression, the plastic strain induces orientation changes of the grains that constitute the polycrystal, leading to a non-random crystallographic texture. The texture can be measured by different diffraction techniques [15] and is a signature of the micromechanical processes that are active during deformation. It is possible to reveal their relative importance by polycrystal plasticity simulations. A very effective modeling approach is the viscoplastic self-consistent (VPSC) polycrystal model, originally introduced by Molinari et al. [16] and later followed by Lebensohn and Tomé [17]. A tuning of the VPSC model was carried out by Molinari and Toth in 1994 [13] using finite element results on the inclusion problem. In the present work, the textures were also measured and the VPSC simulation was carried out for the texture evolution in compression. The obtained results revealed the reason for the deviation from the axisymmetric deformation and the effect of dynamic recrystallization, which appeared to be small on the texture evolution.

## 2. Materials and Experiments

**Figure 1:**
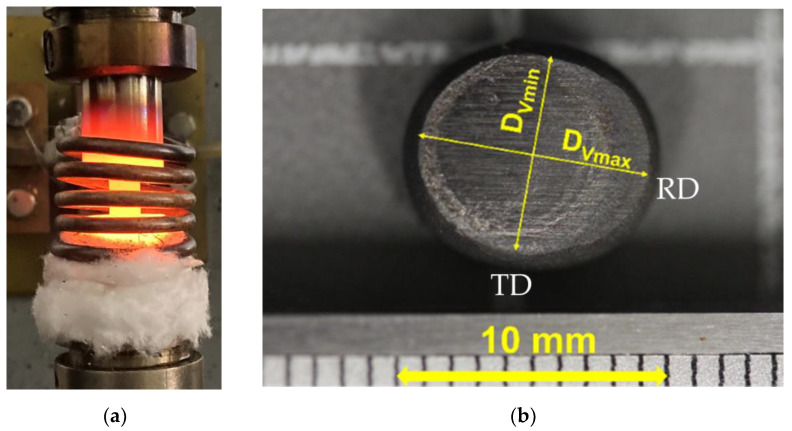
(**a**) Heating of the specimen up to the forming temperature. (**b**) Top view of the compressed material.

Cylindrical specimens with a 6 mm diameter and 8 mm height were machined from a hot-rolled sheet 8 mm thick; the chemical composition was determined using the Oxford Instruments X-MET 8000 XRF Analyzer, displayed in Table 1 (Abingdon, UK). The specimens were compressed between two flat dies at temperatures of 700, 800, 900, and 1000 °C, using an Instron 5982 type material testing machine (Figure 1a) (Norfolk County, MA, USA). The strain rates were set constant at 0.001, 0.01, 0.1, and 1.0 s^−1^. The contact surfaces of the specimens were lubricated with a graphite solid lubricant to reduce friction. During the experimental procedure, the specimens were heated up to the forming temperature with a 5 min holding time before compression. The force and the displacement of the upper die were measured during the whole process. Some barreling and anisotropy in the deformation of the compressed specimens were observed, as presented in Figure 1b. The deformation was larger in the RD direction compared with the TD, about twice as large, as could be estimated from the geometry.

For measuring crystallographic textures, X-ray diffraction patterns were recorded using a Siemens D5000 four circles diffractometer equipped with Co Kα radiation (1.79 Å) (Munich, Germany) and a linear LynxEye detector (Stockholm, Sweden). The pole figures were measured at the positions corresponding to (110), (200), and (211) (hkl) plans. The pole figures were corrected from the measured background for each tilt position.

## 3. Experimental Results

### 3.1. Compression Conditions

Due to the fact that the compressing punches were deformed elastically during testing, and that barreling appeared during the compression process, it was required to correct the experimental data. In the first step, the effect of excess displacements due to the elastic deformation of the punches was eliminated from the displacement data. Then, a correction of the applied pressure was carried out due to the effect of friction that occurred on the two interfaces between the punches and the compressed material, which was responsible for the barreling. Namely, when a specimen with ideal geometry is uniaxially compressed under frictionless conditions, the applied pressure (*p = F/A*) and the von Mises equivalent flow stress ($\bar{\sigma }$) are equal. However, due to the friction of the sample with the die, a larger pressure is needed, leading to the following formula [18]:

(7) $$\bar{\sigma }=\frac{p}{\left(1+\frac{\mu }{3\sqrt{3}}\cdot \frac{d}{h}\right)},$$

where *h* and *d* denote, respectively, the instantaneous height and the diameter of the sample. The above relation can be obtained by the slab method using the Tresca friction law $\tau =\mu \cdot \tau_{k}$, where τ is the friction stress, $\tau_{k}$ is the shear flow stress of the material, and μ is the friction factor (0 ≤ *μ* ≤ 1). If *μ* = 0, the state is frictionless, and *μ* = 1 implies sticking conditions. For the construction of a stress–strain curve, the true compression strain was used with the positive value:

(8) $$\varepsilon =-ln\left(\frac{h}{h_{0}}\right),$$

where $h_{0}$ is the initial height of the specimen. It is difficult to avoid a minimum barreling of a compression specimen, even with good lubrication. Roebuck et al. [19] defined a parameter (*B*), which can characterize the extent of barreling:

(9) $$B=\frac{h\;{R_{max}}^{2}}{h_{0}{R_{0}}^{2}},$$

where $h_{0}$ and $R_{0}$ are the initial height and radius before deformation, respectively, while *h* and $R_{max}$ are the final values after deformation, respectively. When *B* is in the range of 1.0 and 1.1, there is no need for friction correction. In the present work, *B* was relatively high: 1.14, so a correction procedure was applied for the stress values. To determine the average Tresca friction coefficient, the formula proposed by Ebrahimi and Najafizadeh [20] was used:

(10) $$\mu =\frac{\left(\frac{{\bar{r}}_{f}}{h_{f}}\right)b}{\left(\frac{4}{\sqrt{3}}\right)-\left(\frac{2b}{3\sqrt{3}}\right)}\cdots ,\;\mathrm{with}\;\;b=4\frac{∆R}{\bar{r}}\cdot \frac{h}{∆h}$$

**Figure 2:**
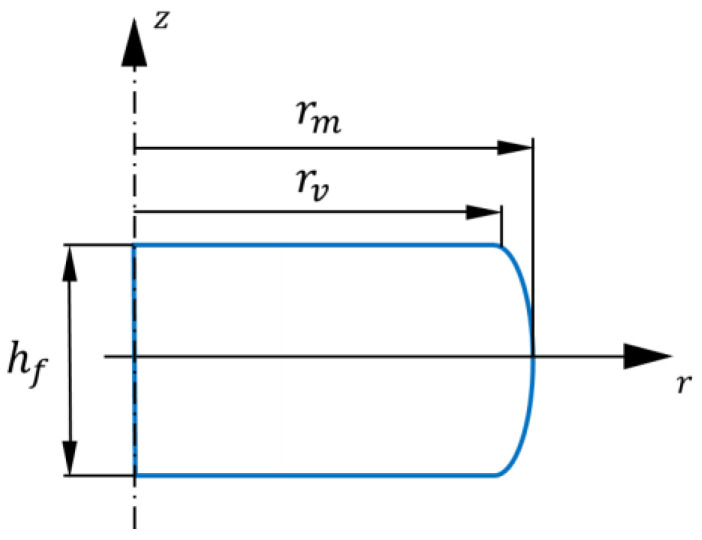
Geometrical parameters of the specimen after compression.

Here, (${\bar{r}}_{f}$) is the geometric mean value of the final radius, $h_{f}$ is the final height, *b* is the so-called barreling shape factor, $\bar{r}$ is the calculated radius in the current position without barreling, ∆*h* is the difference between the initial and final height, and ∆*R* is the difference between the maximum ($r_{m}$) and minimum ($r_{v}$) radii of the specimen after compression, see Figure 2. The obtained average value of *µ* was 0.31 in our experiments.

### 3.2. Grain Structure

**Figure 3:**
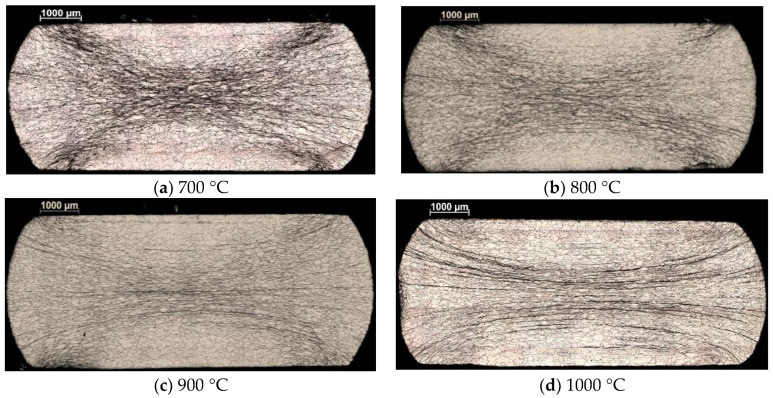
Micrographs of two specimens deformed at 700 °C (**a**), 800 °C (**b**), 900 °C (**c**), and 1000 °C (**d**), with a strain rate of 0.01 s^−1^ to a height reduction of 50%.

The hot-compressed specimens were cut in half along the longitudinal axis to obtain micrographs of the cross-sectional area. After grinding and polishing the samples, their surfaces were chemically etched with the mixture of [HF, HNO_3_, HCL] in the ratio of [3:2:1] to reveal the grain structure. Figure 3a–d show the optical microscopy images of the specimens that were hot compressed at 700 °C, 800 °C, 900 °C, and 1000 °C, at a strain-rate of 0.01 s^−1^. The different elongated nature of the grains and a nonhomogeneous strain field is evidenced in the micrographs. The strongly deformed zones were mainly located in the middle of the cross-section. The region filled with less elongated grains appeared in regions close the contact surface. The 700 °C specimen showed a strong X-shaped deformation localization pattern. In the deformation zone, only elongated grains were dominating, and evidence of a partial dynamic recrystallization process was visible in the samples deformed at higher temperatures. The average grain size was about 200 µm.

### 3.3. Stress–Strain Curves

**Figure 4:**
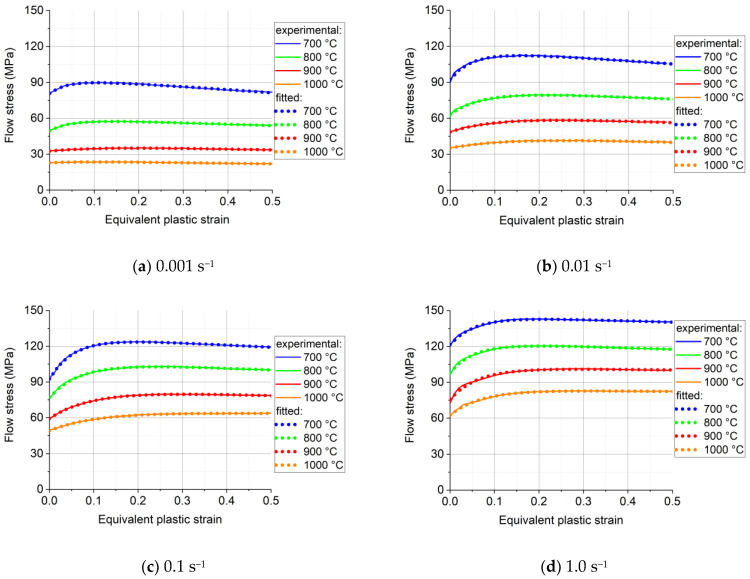
Measured and fitted flow stress–strain curves of Ti57-Nb43: (**a**) 0.001 s^−1^, (**b**) 0.01 s^−1^, (**c**) 0.1 s^−1^, and (**d**) 1.0 s^−1^. The fitted curves were derived from Equation (13).

The measured stress–strain curves after correction for friction and barreling are displayed in Figure 4. The fitted flow curves are also shown in the figure; they will be discussed in Section 5. The flow curves can be divided into two stages. First, the flow stress increases from the initial value up to a peak, which is the work hardening stage. After the peak value, the flow stress starts to decrease gradually with increasing strain; this second stage is a softening stage, which can be attributed to dynamic recrystallization. At larger strains, the flow stress seems to stabilize around a constant value.

**Figure 5:**
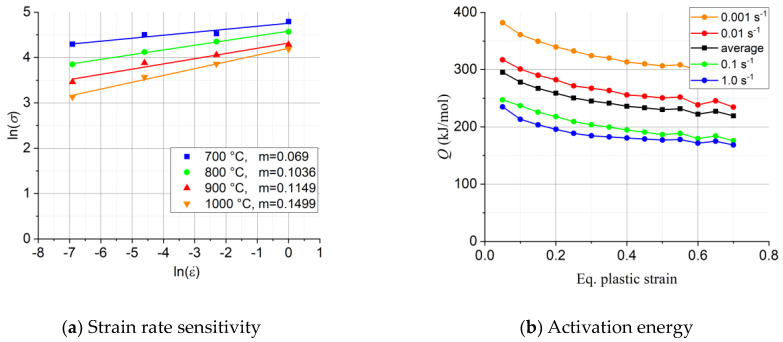
(**a**) Strain rate sensitivity diagram at zero plastic strain. (**b**) Variation of activation energy as a function of the equivalent plastic strain and strain rate.

The stress–strain curves confirm that the Ti57-Nb43 alloy is sensitive to the strain rate at elevated temperatures: the flow stress increases with increasing strain rate. Meanwhile, the flow stress decreases considerably with increasing temperature due to more thermal activation. Figure 5a shows the strain rate sensitivity plot, based on the viscoplastic constitutive relation of Hoff [21]:

(11) $$\sigma =\sigma_{0}{\left(\frac{\dot{\varepsilon }}{{\dot{\varepsilon }}_{0}}\right)}^{m},$$

where σ is the stress at the strain rate $\dot{\varepsilon }$, while $\sigma_{0}$ and ${\dot{\varepsilon }}_{0}$ are their reference values, respectively. *m* is the strain rate sensitivity index, which can be expressed as:

(12) $$m=\frac{d(ln\sigma )}{d(ln\dot{\varepsilon })},$$

Relation (12) is valid at a constant value of the strain. *m* is constant at a given temperature, so the slope of the $ln\sigma -ln\dot{\varepsilon }$ line is equal to the strain rate sensitivity index, see Figure 5a. The *m* values varied between 0.069 and 0.1499. The *m* value at 700 °C seems to be falling off the general trend compared with the values at higher temperatures.

The activation energy (*Q*) is an equally important parameter that can give information on the metallurgical processes taking place in the material. *Q* was calculated according to the Arrhenius expression (Equation (4)), using the procedure described in Ref. [8]: see the result as a function of strain rate and strain in Figure 5b (there was no noticeable dependence of *Q* on temperature). The activation energy varied over a relatively wide range, and it was significantly affected by the plastic strain and the strain rate. An average *Q* value was also calculated and was found to be *Q* = 310.36 kJ mol^−1^, significantly larger than the one previously reported by Wan et al. [9] for the Ti63-Nb37 alloy: *Q* = 230.59 kJmol^−1^.

## 4. Computational Results on Stress–Strain Curves

### 4.1. Voce-Type Constitutive Equation to Describe the Stress–Strain Behavior of Ti57-Nb43

The modified Voce equation is capable of capturing both the hardening and softening stages of the material behavior at elevated temperatures and it has already been successfully applied by Nguyen et al. [5] to describe the stress–strain curves of a titanium alloy in hot deformation:

(13) $$\sigma_{F}(\varepsilon ,\dot{\varepsilon },T)=\sigma_{0}+q_{1}\left(1-exp\left(-c_{1}\varepsilon \right)\right)+q_{2}\left(1-exp\left(c_{2}\varepsilon \right)\right),$$

where $q_{1}$ and $q_{2}$ are the hardening coefficients, $c_{1\;}$ and $c_{2}$ are the softening exponents, and $\sigma_{0}$ is the yield stress. Since the examined titanium–niobium alloy also showed hardening and softening behavior, Equation (13) was selected for simulating the experimental flow curves. A fitting procedure was implemented using the Maple mathematical software (v2019.0) to determine the coefficients of the model function for the tested Ti-Nb alloy. There are five parameters in Equation (13), but only four of them need to be determined by the numerical fitting procedure, because the yield stress, the $\sigma_{0}$ parameter, is known directly from the experiment.

We carried out a fitting procedure with Equation (13) for all measured stress–strain curves that are displayed in Figure 4. However, here we present detailed results only for the measurements that were carried out at the strain rate of 0.001 s^−1^. The reason for this is that we propose in this work a simplification of Equation (13), and the results obtained by reducing the number of variables will be presented later below.

**Figure 6:**
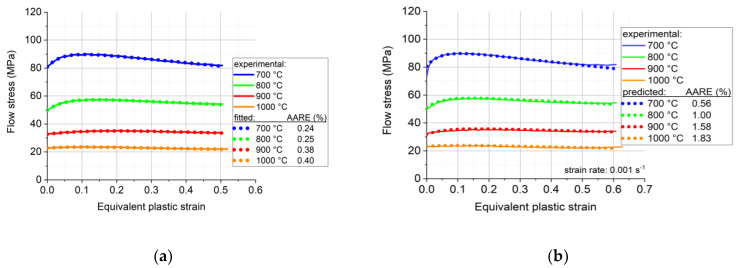
The measured and their fitted curves obtained using Equation (13) at the compression strain rate of 0.001 s^−1^, at four temperatures. (**a**) Separate fit for each curve, and (**b**) fit by using polynomial coefficients for the four parameters, expressed directly as a function of temperature.

Figure 6a displays the result of the fitting procedure with Equation (13) at four temperatures: 700, 800, 900, and 1000 °C, at the strain rate of 0.001 s^−1^. As can be seen, a very good fitting was possible using Equation (13). The corresponding parameter values are displayed in Table 2.

The precision of the fitting is displayed in the inset of Figure 6a, which shows the absolute average relative error (AARE) in percentages. It is defined by the following statistical formula:

(14) $$AARE=\frac{1}{N}\sum_{i=1}^{N}\left|\frac{E_{i}-P_{i}}{E_{i}}\right|\times 100\%,$$

where *N* is the number of measured data points in flow stress $E_{i}$, and $P_{i}$ is the value obtained from the fitting. The quality of the fit is extremely good: the highest deviation is only 0.4%.

**Figure 7:**
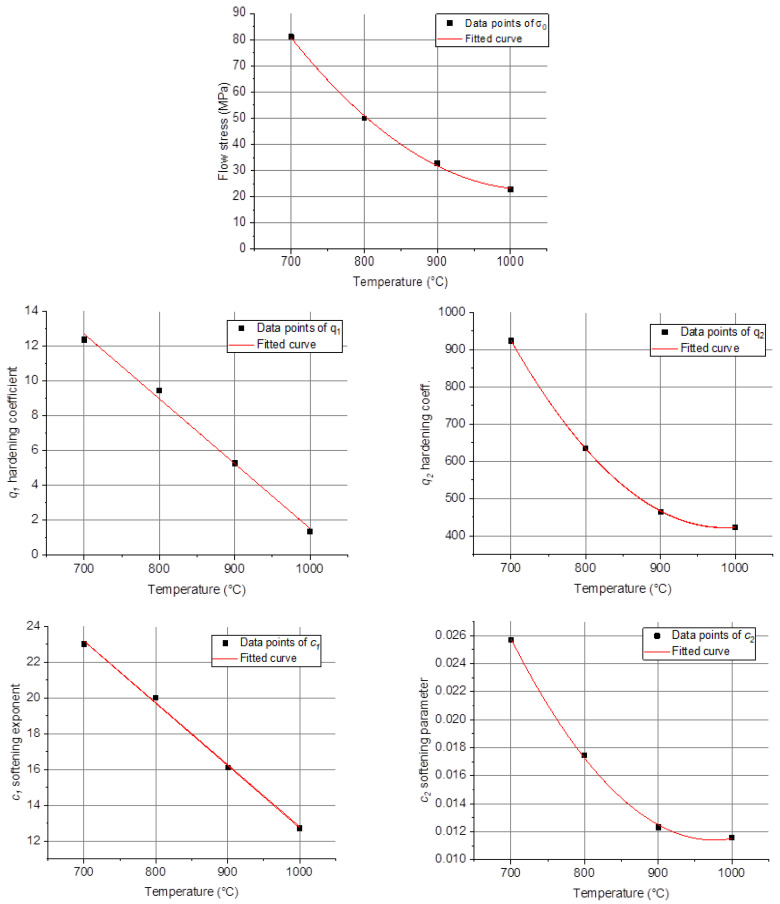
Dependence of the yield stress, $\sigma_{0}$, and the $q_{1}$, $q_{2}$, $c_{1}$, and $c_{2}$ parameters in Equation (13) on temperature, for testing at 0.001 s^−1^, at four temperatures.

It is also possible to fit the $\sigma_{0}$, $q_{2}$, $c_{2},{\;q}_{1}$, and $c_{1}$ parameters by low-order polynomial functions, in order to express them directly as a function of temperature. Such a procedure is actually decreasing the number of parameters, because the same ones are needed for all temperatures. Indeed, $\sigma_{0}$, $q_{2}$, and $c_{2}$ can be described by second-order functions, while for $q_{1}$ and $c_{2}$ simple linear functions are sufficient; they are all shown in Figure 7, while the polynomial coefficients are specified in Table 3. Figure 6b displays the result of the polynomial fitting process. Although the inset in Figure 6b shows that there was some decrease in the AARE values of the fitted curves, they still remained high, and the advantage is that the results are fully analytical: the curves can be obtained for any temperature at the strain rate of 0.001 s^−1^.

### 4.2. Reducing the Number of Parameters

The number of parameters used in a fitting procedure is an important issue; it should be as small as possible. In the following, we propose to use Equation (13) with a reduced number of fitting parameters. Our research in this direction led to the proposition of eliminating the $c_{2}$ parameter, which is taken to 1.0:

(15) $$\sigma_{F}(\varepsilon ,\dot{\varepsilon },T)=\sigma_{0}+q_{1}\left(1-exp\left(-c_{1}\varepsilon \right)\right)+q_{2}\left(1-exp\left(\varepsilon \right)\right),$$

Using the Maple software for such a fitting procedure, the results obtained are presented in Figure 4, for the four temperatures and four strain rates. As can be seen (Table 4), the experimental curves could be approximated with very good precision; the AARE values were between 0.15% and 1.52%.

### 4.3. Analytical Expression of the Parameters as a Function of Temperature and Strain Rate

A convenient expression of parameters that vary as a function of temperature and strain rate is a polynomial form. It permits us to obtain the stress–strain curve at any temperature and strain rate that are within the investigated interval. The efficiency of such modeling was demonstrated above for the Voce parameters in Equation (13), but only for temperature dependence. Here, we present a similar approach that is able to capture at the same time the effect of the temperature and the strain rate in an analytical form, in the reduced parameter approach of Equation (15). For the polynomial, we adopted the following double-variable analytical function composition, where a member of the series has the form:

(16) $$a_{i}x^{j}y^{k}.$$

Here, *x* and *y* are the variables, defined for the temperature and the strain rate, respectively, and the *a* values are the coefficients. The variables were defined as

(17) $$x=1+\frac{T}{T_{ref}},\cdots y=1+\ln\frac{\dot{\varepsilon }}{{\dot{\varepsilon }}_{0}}.$$

Here, $T_{ref}$ and ${\dot{\varepsilon }}_{0}$ are reference values: $T_{ref}=$ 700 °C and ${\dot{\varepsilon }}_{0}=$ 0.001 s^−1^. The following form of the polynomial function was found to perform very well:

(18) $$z\left(x,y\right)=a_{2}y^{2}x^{2}+a_{3}y^{2}x+a_{4}yx^{2}+a_{5}y^{2}+a_{6}x^{2}+a_{7}yx+a_{8}y+a_{9}x.$$

This polynom contains eight coefficients, which had to be determined for each parameter in Equation (15), for $\sigma_{0}$, $q_{1}$, $c_{1}$, and $q_{2}$.

**Figure 8:**
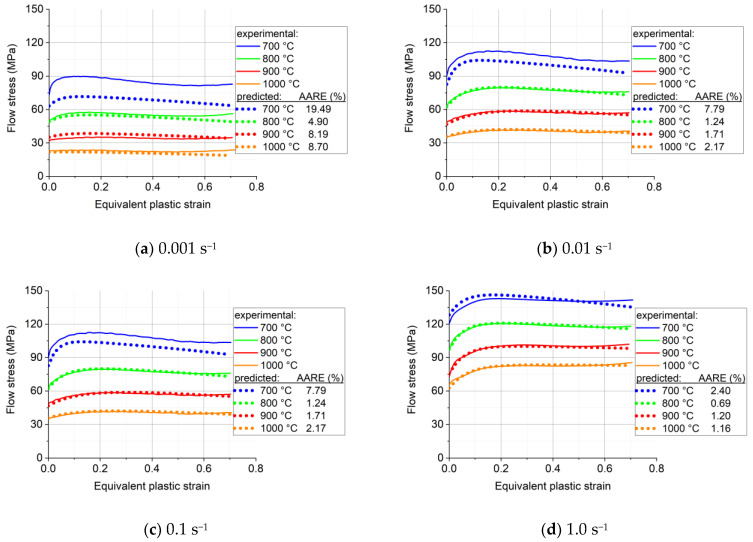
Measured and polynomial-predicted stress–strain curves using the function defined in Equation (15) for the four strain rates and four temperatures.

**Figure 9:**
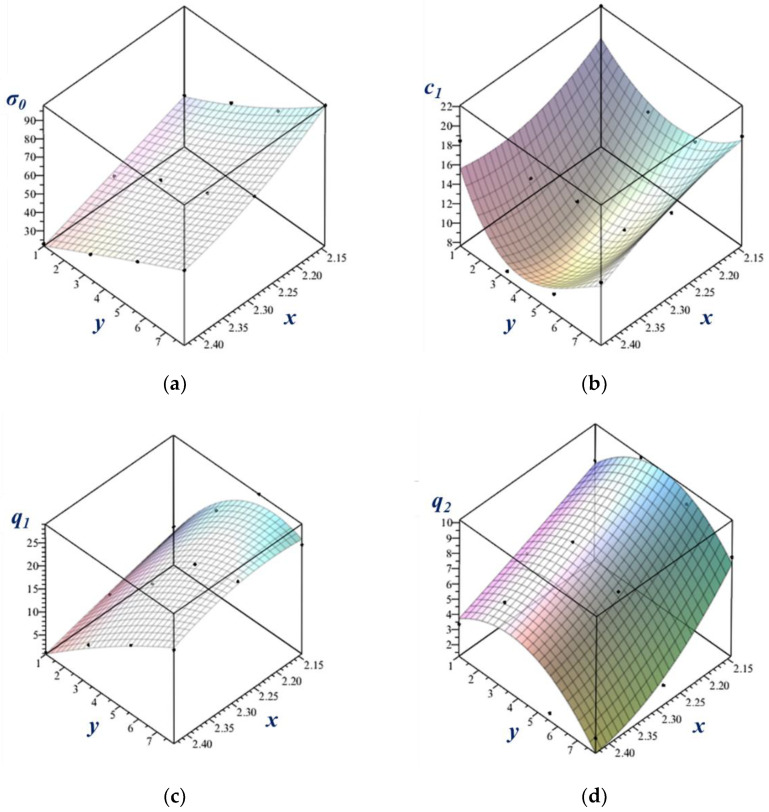
(**a**–**d**) Fitted surfaces on the data points of the material parameters as a function of the strain rate and temperature.

The result of the Maple fitting process is shown in Figure 8, and the coefficients are displayed in Table 5. Figure 9 shows the data points of the material parameters together with the fitted surfaces as a function of the *x* and *y* variables.

## 5. Texture Measurements and VPSC Modeling Results

**Figure 10:**
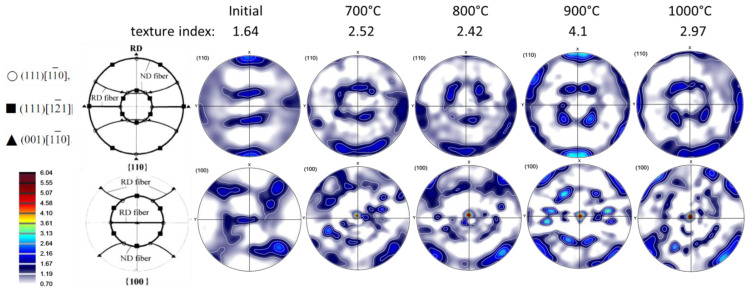
Measured textures shown in (110) and (100) pole figures. The ideal fibers of b.c.c. rolling textures are also shown with the identification of three major ideal orientations [23].

The raw pole figure measurements were processed to obtain the recalculated ones using the ATEX software (v.4.10) [22]. The measured crystallographic textures of the initial material and that of the samples deformed at 700, 800, 900, and 1000 °C at the compression strain rate of 0.1 s^−1^ are presented in (110) and (100) pole figures in Figure 10. The phase is b.c.c., for all temperatures in this alloy. The initial state of the material was a hot-rolled plate, from which cylindrical-shaped samples were cut out in the normal direction of the plate. Therefore, the initial state of the material was a rolling texture (Figure 10). Because a texture already existed in the material before testing, the original RD, TD, and ND directions were tracked on the samples for all texture measurements, and the textures are presented in that reference system. The ideal orientations of b.c.c. rolling textures are also presented in the (110) and (100) pole figures in Figure 10 (from Ref. [23]). As can be seen from the pole figures, the initial texture contained the two major ideal fibers of rolling, called RD and ND fibers, which are defined by <110> parallel to RD and <111> parallel to ND, respectively. Nevertheless, these fibers did not present uniform intensities; three ideal orientations were preferred: the $\left(111\right)[1\bar{1}0]$, the $\left(111\right)[1\bar{2}1]$, and the $\left(001\right)[1\bar{1}0]$, identified in the key pole figures. After deformation, the texture changed slightly: the ND fiber became more uniform, while, along the RD fiber, the $\left(001\right)[1\bar{1}0]$ component became the major texture component. This component is an ND-45° rotated cube position. There are also noticeable variations in the strength of the textures as a function of processing temperature; the texture index is indicated in Figure 10. The initial texture is actually quite weak with its index of 1.64 (for a random texture it is 1.0). There is some strengthening due to the deformation at 700 and 800 °C up to about a value of 2.5. The texture is the strongest at 900 °C, where it displays an index of 4.1. At 1000 °C, its intensity is 2.97. One can see in Figure 10 that the textures are slightly rotated around the TD axis at 700, 800, and 1000 °C, which can be due to some misalignment in the sample positions; the texture seems the most symmetric at 900 °C. Note that the textures were processed without applying sample symmetries. Such an operation would reduce the texture intensity and could only be applied if the initial texture and deformation were symmetric, and, moreover, if the grain size was sufficiently small for good statistics. While the initial texture and compression were relatively symmetric, the grain sizes were not small enough at high temperatures due to recrystallization (about 200 µm), so some spotty nature of the measured textures can be seen in the pole figures.

The VPSC polycrystal simulations were carried out using the finite element calibrated version of the Metz-VPSC code; see Ref. [13] for the modeling approach. The value of the so-called interaction parameter (α in the localization equation of the VPSC model) was chosen to be very low (0.01), meaning that the stress states in the grains of the polycrystal were nearly equal to the macroscopic one (‘static’ approach). As the material was in a b.c.c. state, the 12 $\left(110\right)[1\bar{1}1]$ and 12 $\left(112\right)[11\bar{1}]$ slip systems were used as possible slip systems with equal strengths. The strengths of the slip systems were kept constant during the simulation, i.e., strain hardening was not simulated. This was done in order to examine only the effect of the evolution of the texture on the compression stress–strain curve. The value of 0.1 was used for the viscoplasticity index of slip (the ‘m’ parameter), which is the average value of the measured strain rate sensitivities at high temperatures of this alloy (see Figure 5a). The initial texture was discretized by the ATEX software to 5000 grain orientations and used for the compression simulation. The macroscopic imposed strain rate tensor was imposed without shear components, and the relative values of the three normal strain components were set according to the experimental observation. Namely, as mentioned above in the experimental part, the cross-section of the cylindrical samples became elliptic (see Figure 1b), so that the strain ratio between the RD and TD strains was about 2.0. This situation was imposed in the simulation. The VPSC calculation was carried out in 50 small strain increments with an increment size of 0.01 to obtain the final true compression strain of −0.5.

**Figure 11:**
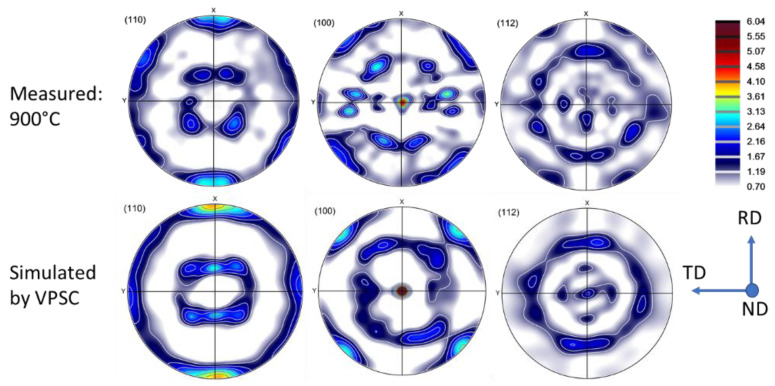
Measured and simulated textures in hot compression of Ti-Nb in three pole figures ((110), (100), and (112)).

The simulated textures are displayed in Figure 11 in three pole figures ((110), (100), and (112)), in comparison with the experimental texture measured at 900 °C. As the textures were similar at all temperatures, only one simulation was carried out. There is a good agreement between the simulation and experiment, and even some of the details are reproduced by the simulation. Concerning the evolution of the compression stress as a function of strain, only a slight increase was predicted by the VPSC model: up to 1.5% in flow stress.

## 6. Discussion

As was pointed out in the Introduction, the analytical description of the stress–strain response of the investigated alloy is important for the forming processes that are needed for its use in applications. This alloy was plastically deformed at high temperatures where dynamic recrystallization can take place. Therefore, its stress–strain behavior is complex, and includes strain hardening as well as softening stages. The mathematical description of such curves requires several parameters that vary when the temperature and the strain rate are changed. It is important to describe the curves with a minimum number of coefficients. The modified Voce equation (Equation (13)), presented by Nguyen et al. [5], is a very suitable function for this purpose, and its applicability for the experiments carried out in this work has been demonstrated. One of the most important results of the present work is that the number of parameters of the modified Voce equation can be reduced from five to four for the case of compression of the Ti-Nb alloy. The other major result is the approximation of the four coefficients by a suitable polynom, with two variables, that are the temperature and the strain rate. One advantage of such a procedure is a further reduction in the number of coefficients. Indeed, for the present experiments, where four temperatures and four strain rates were considered, the total number of coefficients was 4 × 4 × 4 = 64. For the polynomial description, it was only 4 × 8 = 32. The other advantage is that the polynom can be used at any temperature and strain rate, while the non-polynomial approach is only valid for the 4 × 4 measured cases, so does not have a predictive capacity.

Nevertheless, it is also true that the polynomial approach was not applicable to the whole investigated temperature and strain rate ranges. It was found that it was not working well for the lowest temperature (700 °C), and for the lowest strain rate (0.001 s^−1^). From an industrial point of view, the very low strain rate is not an issue because industrial plastic forming processes are carried out at high strain rates. We have actually examined material behavior up to 1.0 s^−1^, which is in the range of industrial applications. Concerning the deviation at 700 °C, it can be explained by the strong strain localization in form of an X, well visible on the metallographic image in Figure 3a. Such localization is due to the unstable nature of the deformation at that temperature, which is to be avoided, so the forming operation should be carried out at higher temperatures. At the same time, the strain rate sensitivity parameter, m, was found to be relatively low and out of trend at 700 °C, which could have also contributed to the localization of the deformation. It is well known that a higher strain rate sensitivity delays strain localization, so forming at high temperatures, where the strain rate sensitivity is high, is advantageous for formability. Note that Sarkar et al. [24] also observed strain localization below 1000 °C in the Nb-1Zr-0.1C alloy, which led to two distinctly different domains under the conditions of hot deformation.

**Figure 12:**
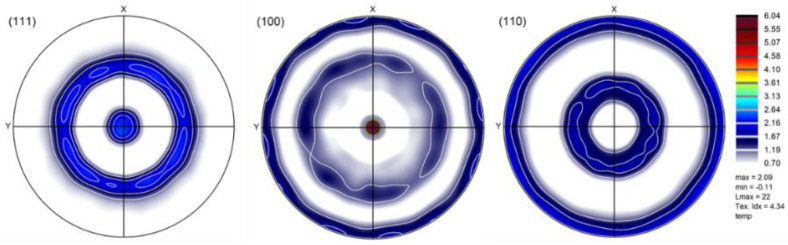
Simulated texture for an axisymmetric compression of −0.5 using a random initial texture represented by 5000 grain orientations, displayed in (111), (100), and (110) pole figures.

The texture measurements together with the VPSC modeling were helpful for clarifying the role of the texture in the behavior of the material during compression. Two effects were examined: the shape change and the strain hardening. Due to the previous hot rolling of the samples, there was a rolling texture in the material before compression. Therefore, the strain response was not the same in the RD and TD directions, leading to twice more strain in the RD direction than in the TD direction. This effect led to an elliptical shape of the cross-section of the sample, so the texture played an important role in the deformation. To obtain information on the role of the texture in the strain hardening behavior of the alloy, it was only possible by carrying out polycrystal plasticity simulations for which purpose the VPSC model was used. VPSC modeling provides two important results: the material strength and the evolution of the crystallographic texture. Once the simulation is reproducing the experimental texture well, the effect on the strain hardening is also correctly obtained. It was found in the present compression experiments that there was no significant effect of the evolution of the texture on the flow stress of the material; only about a 1.5% increase was observed during the compression strain of 0.5. As softening was not obtained by the simulation, only a 1.5% hardening, it means that dynamic recrystallization must have been responsible for the decrease in the flow stress at larger strains at higher temperatures. The changes in the texture between the initial state and the final deformed state were also not significant, both in the simulation and in the experiments. This can be understood by examining the texture changes during deformation, which were found to be relatively small. Indeed, the strain mode did not change dramatically when compression was applied after rolling. The strain rate tensors (***D***) for compression and for rolling were the following:

(19) $$\begin{array}{c} D_{compression}=\left\|\dot{\varepsilon }\right\|\cdot \left(\begin{array}{ccc} 0.67 & 0 & 0\\ 0 & 0.33 & 0\\ 0 & 0 & -1.0 \end{array}\right),\;\\ D_{rolling}=\left\|\dot{\varepsilon }\right\|\cdot \left(\begin{array}{ccc} 1.0 & 0 & 0\\ 0 & 0.0 & 0\\ 0 & 0 & -1.0 \end{array}\right) \end{array}$$

where $\dot{\varepsilon }$ is the compression strain rate. The relatively small difference led to a small evolution of the texture, so the strength was not influenced significantly, but it could be still characterized by the texture components of rolling. It is important to point out that, if the initial texture was random (isotropic), the simulation could not produce the observed experimental texture. Namely, in such a case, the deformation behavior of the initially isotropic material would lead to axisymmetric compression, i.e., the sample cross-section would have remained circular, not elliptic. Also, the simulated texture deviated significantly from the experimental one if a random initial texture was used, see the result in Figure 12, where the texture can be characterized by two ideal fibers: <001>||ND and <111>||ND. A comparison of this predicted texture with the simulated and experimental ones displayed in Figure 11 shows clearly that it is important to consider the anisotropy of the material before compression achieve good modeling of the texture evolution.

## 7. Conclusions

The high-temperature deformation behavior and the evolution of the crystallographic texture of the Ti57-Nb43 alloy were investigated in this study in hot compression testing in the temperature range of 700–1000 °C and the strain rate range of 0.001–1.0 s^−1^. The obtained experimental results and their analysis led to the following major conclusions:

- The stress–strain curves of the examined Ti-Nb alloy can be very well approximated by the modified Voce equation proposed by Nguyen et al. [5] using five parameters.
- It has been shown that the number of fitting parameters could be reduced to four in the Nb-Ti alloy.
- A polynomial two-variables function is proposed to express the four parameters as a function of temperature and strain rate, which could well capture the stress–strain curves in the temperature and strain rate range of 800–1000 °C and 0.01–1.0 s^−1^, respectively. The results for 700 °C deviated because of strain localization.
- The experimental crystallographic texture was faithfully reproduced by VPSC polycrystal modeling by considering the initial rolling texture in the material.
- The VPSC modeling showed that the texture evolution affected only 1.5% of the evolution of the flow stress during compression, so its effect on the strain hardening characteristics was practically negligible. However, the initial anisotropy was responsible for the elliptical shapes of the cross-sections of the specimens.

## Figures and Tables

**Table 1:** The chemical composition of the Ti57-Nb43 alloy (weight %).

Ti	Nb	Au	Fe	Pd	Mn	rest
56.54	42.81	0.18	0.12	0.12	0.06	0.16

**Table 2:** The parameter values of Equation (13) for the measurements carried out at 0.001 s^−1^.

Parameter Values	Temperature (°C)			
	700	800	900	1000
$\sigma_{0}$ [MPa]	80.84	49.76	32.62	22.75
$q_{1}$ [MPa]	12.36	9.433	5.268	1.343
$q_{2}$ [MPa]	924.0	634.2	464.7	422.9
$c_{1}$	23.01	20.01	16.10	12.71
$c_{2}$	0.0257	0.01743	0.01231	0.01156

**Table 3:** The polynomial coefficients for fitting the parameters of Equation (13) as a function of temperature for the measurements carried out at 0.001 s^−1^.

Parameters	Polynomial Coefficients		
	Constant	Linear Coefficient	Second-Order Coefficient
$\sigma_{0}$ [MPa]	584.9068	−1.0905	5.2884 × 10^−4^
$q_{1}$ [MPa]	38.7562	−0.0372	0
$q_{2}$ [MPa]	6436.6547	−12.216	0.0062
$c_{1}$	47.4508	−0.0346	0
$c_{2}$	0.1906	−3.6709 × 10^−4^	1.8798 × 10^−7^

**Table 4:** Fitted values of the parameters in Equation (15) at four temperatures and four strain rates.

Strain Rate	Temperature	*σ*_0_	*q*_1_	*c*_1_	*q*_2_	AARE
[s^−1^]	[°C]	[MPa]	[MPa]		[MPa]	[%]
0.001	700	80.773	11.531	26.211	17.109	1.52
	800	49.745	9.107	22.132	7.776	0.27
	900	32.638	4.064	9.465	4.837	0.39
	1000	22.771	1.257	18.480	3.334	0.52
0.01	700	92.430	23.544	20.833	16.459	0.35
	800	63.451	19.091	14.618	10.097	0.34
	900	48.654	12.790	10.499	7.699	0.33
	1000	35.192	9.343	8.430	6.898	0.38
0.1	700	92.478	34.473	19.044	12.130	0.15
	800	76.974	29.011	14.955	9.131	0.25
	900	59.322	23.527	10.981	6.547	0.18
	1000	49.209	15.676	9.524	1.759	0.21
1.0	700	121.570	23.264	18.349	7.097	0.21
	800	98.099	24.455	18.944	7.757	0.28
	900	75.721	26.230	16.174	2.538	0.59
	1000	62.656	21.128	14.144	2.232	0.31

**Table 5:** Estimated coefficients of the polynomials.

Params.	*σ*_0_ (*x,y*)	*q*_1_ (*x,y*)	*c*_1_ (*x,y*)	*q*_2_ (*x,y*)
*a*_2_	−3.27538	0.0	−13.46825	0.0
*a*_3_	13.95439	2.011577	62.19368	0.10279
*a*_4_	59.76368	−12.28321	102.38188	8.67384
*a*_5_	−14.60045	−4.96277	−71.1329	−0.44966
*a*_6_	−49.61801	−4.02339	−6.81293	−5.871106
*a*_7_	−267.96893	39.68338	−474.14841	−41.70084
*a*_8_	304.47517	−20.23071	543.23392	51.54085
*a*_9_	126.90537	8.62717	24.51490	15.28197

## Data Availability

Data supporting reported results are available from the authors.
